# Immunoregulation of Shenqi Fuzheng Injection Combined with Chemotherapy in Cancer Patients: A Systematic Review and Meta-Analysis

**DOI:** 10.1155/2017/5121538

**Published:** 2017-01-05

**Authors:** Yang Yang, Wang Ting, Liu Xiao, Fu Shufei, Tan Wangxiao, Wang Xiaoying, Gao Xiumei, Zhang Boli

**Affiliations:** ^1^State Key Laboratory of Modern Chinese Medicine, Tianjin University of Traditional Chinese Medicine, Tianjin 300193, China; ^2^College of Traditional Chinese Medicine, Tianjin University of Traditional Chinese Medicine, Tianjin 300193, China

## Abstract

*Background*. Immunosuppression is a well-recognised complication of chemotherapy in cancer patients. We assemble the clinical evidence that SQI, an adjuvant drug for lung cancer and gastric cancer which was widely prescribed in China, interventions could increase objective tumour response and regulate immunity in cancer patients undergoing chemotherapy.* Methods.* We undertook a systemic review of the clinical data from randomised controlled trials up to September 2015 in which a SQI intervention was compared with a control arm in patients undergoing conventional chemotherapy. Revman 5.0 Software was used for the data analysis.* Results.* 49 randomised controlled trials were included in the systematic review. The meta-analysis results demonstrated that the SQI intervention with conventional chemotherapy exhibited better therapeutic efficacy than the conventional chemotherapy group with a statistically significant higher objective tumour response. Cotreatment with SQI could enhance NK, CD_3_^+^, CD_4_^+^ level, and CD_4_^+^/CD_8_^+^ ratio comparing with the conventional chemotherapy group.* Conclusions.* The conclusions of this review might suggest a high risk of bias due to the low quality and the limitation of cancer types in the included trials. A more reliable conclusion regarding the immunoregulation of SQI could be reached based on more trials of higher quality.

## 1. Introduction

The prevalence of cancer continues to increase globally. Although the mortality of cancer has been reduced through advances in treatment such as chemotherapy, the adverse reactions caused by chemotherapy such as cardiotoxicity, myelosuppression, and immunosuppression have increased [1]. It has been increasingly recognised that alternative medicines might be another strategy, and western medicines might not be the only answer while these issues remain unsolved [2–5].

Shenqi Fuzheng injection (SQI) is an injection comprised of* Codonopsis pilosula (Franch)* Nannf. and* Astragalus membranaceus (Fisch.)* Bunge [6] and was approved by the State Food and Drug Administration of the People's Republic of China (SFDA) in 1999. As an adjuvant drug for lung cancer and gastric cancer, its efficacy is shown in tonifying qi and strengthening the body's resistance. Researches indicated that SQI could improve the peripheral blood T cell subsets, promote macrophage proliferation, and alleviate immunosuppression caused by chemotherapy [7, 8]. Currently, there are many published trials about SQI combined with chemotherapy for the treatment of cancers; some of these trials have shown that SQI could improve tumour response and increase immunity indicators [7–10]. However, little is known about SQI outside of China, and there has not been a systematic evaluation on its effects on immunity until now. The hypothesis of this paper was SQI, an adjuvant drug for lung cancer and gastric cancer which was widely prescribed in China, could make a critical difference in alleviating chemotherapy-associated immunosuppression.

This paper presents a systematic review in an effort to clarify if SQI in combination with conventional chemotherapy for cancer patients increases the objective tumour response and relative immunity parameters.

## 2. Materials and Methods

### 2.1. Search Strategy

According to guidelines from the Cochrane collaboration [11], a literature search of PubMed, CNKI (China national knowledge infrastructure, http://www.cnki.net/), VIP (Chongqing VIP Information Co., Ltd, http://www.cqvip.com/), and Wanfang (http://www.wanfangdata.com.cn/) from 1999 (SQI launch) to September 2015 was performed. The search strategy “((((cancer) OR tumour)) AND shenqi fuzheng injection) AND immune” was adapted for each database. Papers were limited to clinical research in Chinese or English.

### 2.2. Inclusion and Exclusion Criteria

The studies were included if (1) the study was a randomised controlled trial comparing a SQI plus chemotherapy treatment group with a chemotherapy control group; (2) the patients were diagnosed as having cancer with the age, gender, race, cancer type, and pathological classification and chemotherapy regimens were unlimited; (3) the invention was SQI intravenous drip infusion on the basis of conventional chemotherapy adopted by the control group; the initial time, dosage, and course of medicine treatment were unrestricted; (4) studies contained at least one of the following clinical data points: objective tumour response (the 4-point WHO scale was adopted [12]), natural killer cell (NK), matured T lymphocytes (CD_3_^+^), inducer lymphocyte/helper T lymphocyte (CD_4_^+^), suppressor T cell/cytotoxic T cell (CD_8_^+^) level, and CD_4_^+^/CD_8_^+^ ratio; (5) the reported data included estimated relative risk (RR) and 95% confidence intervals (CIs) for each outcome; (6) in the case of duplicate publications, the maximum sample size version was included.

Studies were excluded if they met any of the following criteria: (1) the studies were case series, case reports, or clinical reports concerning radiotherapy or surgery; (2) the paper used SQI in combination with other herbal medicines or chemical drugs; (3) the articles exhibited no outcomes concerning objective tumour response and immunity index or were presented as an abstract only.

### 2.3. Data Extraction and Methodological Quality Assessment

Data were independently extracted by two reviewers (Y. Y. and W. T.) using a data collection table. All discrepancies were resolved by consensus. For the systematic review, all data on patient characteristics (number, gender, age, and oncological category), treatment and invention details (chemotherapy regimens, schedule, and course of SQI invention), and clinical outcomes were extracted. The following outcomes were extracted: objective tumour response and immunity indicators including NK, CD_3_^+^, CD_4_^+^, and CD_8_^+^ levels, and CD_4_^+^/CD_8_^+^ ratio. The quality of the studies included in the analysis was assessed independently by two reviewers (Y. Y. and W. T.). The methodological quality of the studies was assessed using the modified Jadad scale, an instrument developed and validated to assess the quality of clinical trials by evaluating randomization, blinding, withdrawals/dropouts, and randomization concealment [13, 14].

### 2.4. Data Synthesis and Statistical Analysis

Heterogeneity between studies was assessed by measuring inconsistency (*I* ^2^). When *I* ^2^ < 50%, the fixed-effects model was used to calculate the relative ratio (RR) and the 95% confidence intervals (CIs). Otherwise, a random-effects model was used [15]. The publication bias was examined by using funnel plots. A forest plot was built to show the overall effect of the intervention against control. Statistical analyses were performed using RevMan 5.0 (Cochrane Information Management System, Oxford, United Kingdom (UK)) [11], and *P* < 0.05 was considered statistically significant.

## 3. Results

### 3.1. Description of Studies

**Figure 1:**
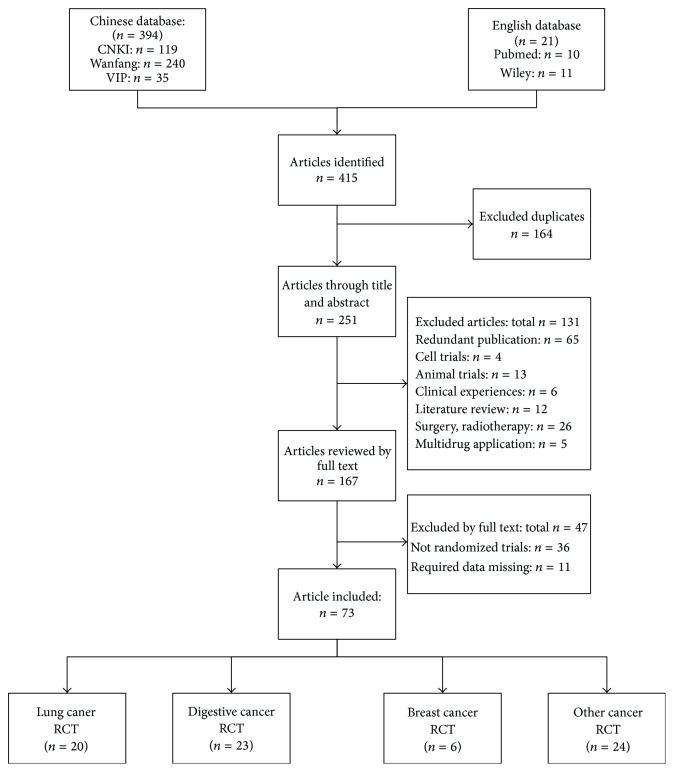
Flow chart of literature screening.

A total of 415 studies were identified through the search of databases. 251 studies were retained after the first screening based on the title and abstract. A total of 131 studies were excluded according to the inclusion and exclusion criteria. Among the studies that were retained, 73 randomised controlled trials were selected after full-text assessment. Forty-nine of the 73 studies were classified into three main categories: 20 trials of lung cancer [16–34], 23 trials of digestive tract cancer [35–56], and 6 trials of breast cancer [8, 57–61] as shown in Figure 1. For lung cancer, 20 trials included 1597 patients with a median age ranging from 43 to 66.5. A dominance of non-small cell lung cancer existed (18/20, 90%), and the small cell lung cancer accounted for 10%. Platinum-based chemotherapy represented by paclitaxel plus cisplatin was the primary chemotherapy (10/20, 50%). Other chemotherapy regimens contain vinorelbine plus cisplatin, gemcitabine plus cisplatin, and docetaxel plus cisplatin. Regarding digestive tract cancer, 23 studies consisted of 1656 patients with the median age range of 45 to 65.9. Colon cancer, colorectal cancer, gastric cancer, gastrointestinal cancer, and oesophageal cancer were all included in digestive tract cancer. Oxaliplatin and 5-Fu based chemotherapy regiments were widely used in clinic. Six articles were focused on breast cancer with 1656 female patients in a median age of 42 to 56.1. Anthracycline-based chemotherapy was the conventional chemotherapeutic agent. According to the modified Jadad scale [14], the methodology of all studies was low quality with a quality score of 3 or under 3. All the clinical details of the 49 included studies were listed in Table 1. The remaining 24 studies which included 10 kinds of cancers like leukaemia, cervical cancer, and ovarian cancer were not included in the meta-analysis because of the lack of samples.

### 3.2. Safety Evaluation of Combination Medication of SQI and Chemotherapy

All articles included in the meta-analysis evaluated the safety of the combination medication of SQI and chemotherapy regiments. Detailed safety evaluation information on the combination medication of SQI and chemotherapy agents showed in Table 2. The conclusion could be drawn from the table that gastrointestinal reactions and routine blood indexes decreases were the primary and most mentioned phenomena.

### 3.3. The Results of Meta-Analysis for Clinical Outcomes in Lung Cancer Patients: Objective Tumour Response and Immunity Indicators

In 12 clinical trials concerning objective tumour response in lung cancer patients [16, 17, 19, 21–25, 28–30, 34], there were 406 patients in the SQI intervention group and 399 patients with conventional chemotherapy in the control group. The results showed that the objective tumour response in the SQI intervention group was better than in the control group (RR = 1.28, 95% CI 1.09–1.49, *P* = 0.002).

**Figure 2:**
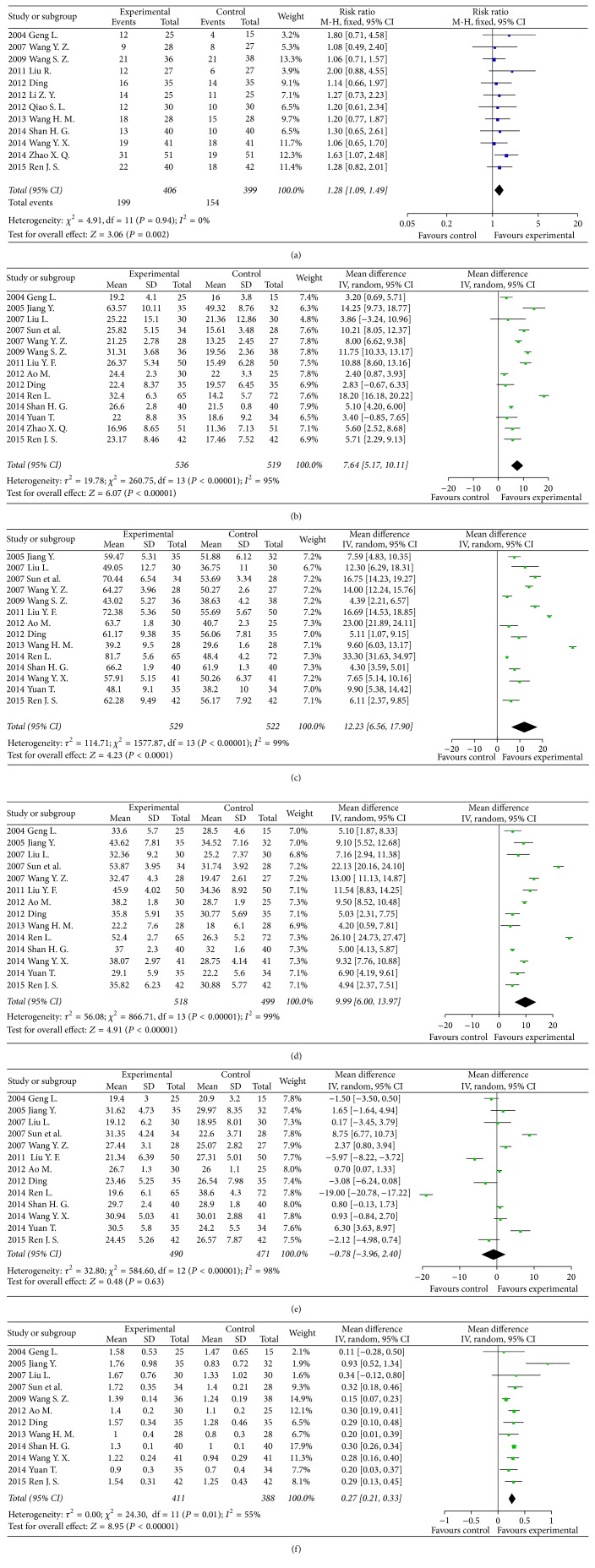
Forest plots of studies comparing Shenqi Fuzheng injection (SQI) invention groups and control groups, measuring the effect of SQI on lung cancer patients including objective tumor response (a) and immunity indicators: NK (b), CD_3_^+^ (c), CD_4_^+^ (d), CD_8_^+^ (e) level, and CD_4_^+^/CD_8_^+^ ratio (f).

According to 14 clinical trials [16–18, 20, 21, 25–27, 29–31, 33, 34] including 536 patients in the SQI intervention group and 519 lung cancer patients with conventional chemotherapy as control group, the NK levels were significant improved by SQI intervention (RR = 7.64, 95% CI 5.17–10.11, *P* < 0.00001). In 14 clinical trials [16, 18–22, 25–27, 29–31, 33] including 529 patients in the SQI intervention group and 522 patients with conventional chemotherapy, CD_3_^+^ cell levels were dramatically improved by SQI (RR = 12.23, 95% CI 6.56–17.90, *P* < 0.0001). For the CD_4_^+^ cell levels in lung cancer, there were 14 clinical trials [16, 18–22, 25–27, 30, 31, 33, 34] including 518 patients in the SQI intervention and 499 patients with conventional chemotherapy. SQI intervention preceded the control group in improving the CD_4_^+^ cell levels with RR = 9.99, 95% CI 6.00–13.97, *P* < 0.0001. There were 12 clinical trials [16, 18, 19, 21, 22, 25, 26, 29, 31, 33, 34] including 411 patients in SQI intervention and 388 patients with conventional chemotherapy mentioned about the CD_4_^+^/CD_8_^+^. The results showed that the SQI intervention group was superior to the control group in improving the CD_4_^+^/CD_8_^+^ ratio (RR = 0.27, 95% CI 0.21–0.33, *P* < 0.00001). Thirteen trials mentioned about CD_8_^+^ cell levels. However, no statistical significance appeared between 490 patients in the SQI intervention group and 471 patients with conventional chemotherapy. The details concerning the results of the meta-analysis for clinical outcomes in lung cancer patients were illustrated in Figure 2.

### 3.4. The Results of Meta-Analysis for Clinical Outcomes in Digestive Tract Cancer Patients: Objective Tumour Response and Immunity Indicators

Regarding the objective tumour response in digestive tract cancer, there were 11 studies including 397 patients in the SQI intervention group and 403 patients with conventional chemotherapy [37–41, 43, 44, 47, 54, 56]. The objective tumour response in the SQI intervention group was better than control (RR = 1.32, 95% CI 1.15–1.52, *P* < 0.0001).

**Figure 3:**
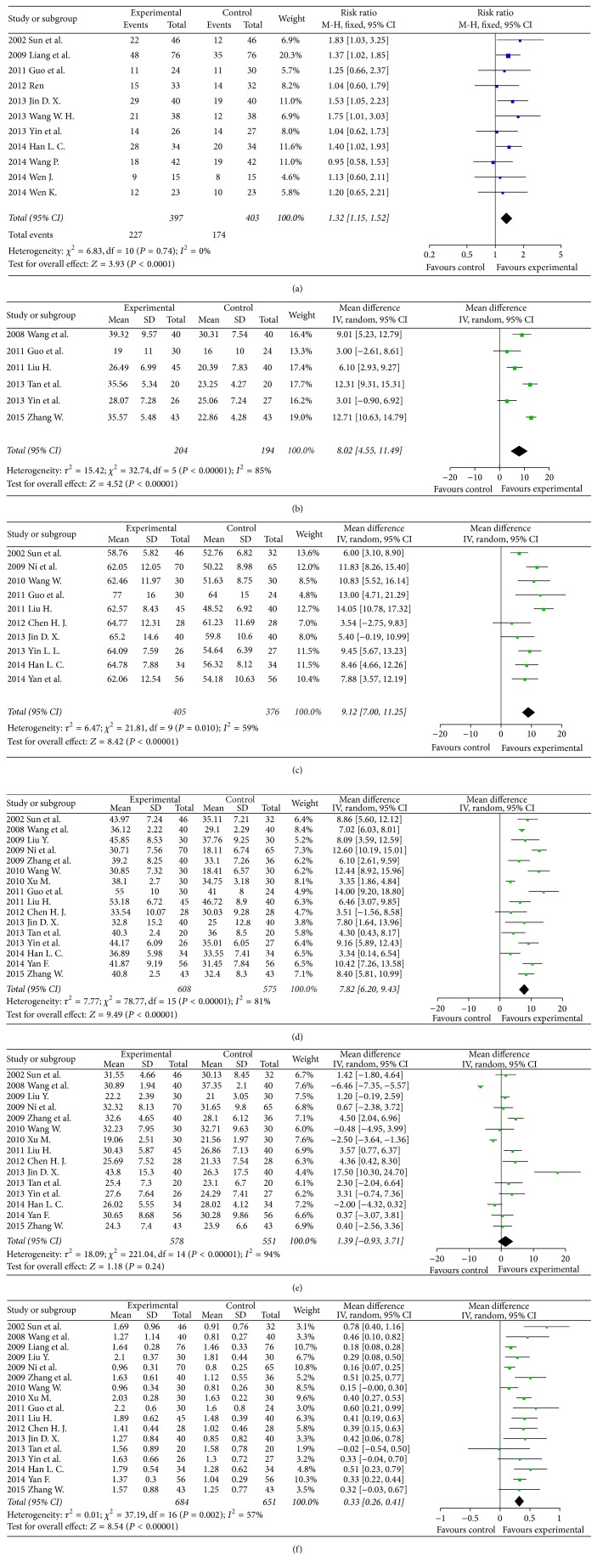
Forest plots of studies comparing Shenqi Fuzheng injection (SQI) invention groups and control groups, measuring the effect of SQI on digestive tract cancer patients including objective tumor response (a) and immunity indicators: NK (b), CD_3_^+^ (c), CD_4_^+^ (d), CD_8_^+^ (e) level, and CD_4_^+^/CD_8_^+^ ratio (f).

Regarding the NK level variations in digestive tract cancer, there were 6 clinical trials [35, 40, 42, 46, 47, 55] including 204 patients in the SQI intervention group and 194 patients with conventional chemotherapy as control. SQI could significantly improving the NK levels versus control (RR = 8.02, 95% CI 4.55–11.49, *P* < 0.00001). In 10 clinical trials [36, 39, 40, 43, 45–47, 50, 52, 56] including 405 patients in the SQI intervention and 376 patients with conventional chemotherapy, the CD_3_^+^ cell levels in digestive tract cancer were statistically significant improved by SQI (RR = 9.12, 95% CI 7.00–11.25, *P* < 0.0001). SQI could also improve CD_4_^+^ cell levels according to 16 trials [35, 36, 39, 40, 42, 43, 45–47, 49–53, 55, 56] including 608 patients in the SQI intervention and 575 patients with conventional chemotherapy (RR = 7.82, 95% CI 6.20–9.43, *P* < 0.0001). The CD_4_^+^/CD_8_^+^ ratio was improved by SQI in 16 clinical trials [35, 36, 39, 40, 42, 43, 45–47, 49–56] which include 684 patients in the SQI intervention and 651 patients with conventional chemotherapy (RR = 0.33, 95% CI 0.26–0.41, *P* < 0.0001). Sixteen trials mentioned about CD_8_^+^ cell levels. There was no statistical significance between 608 patients in the SQI intervention group and 575 patients with conventional chemotherapy. The results of the meta-analysis for clinical outcomes in digestive tract cancer patients were illustrated in Figure 3.

### 3.5. The Results of Meta-Analysis for Clinical Outcomes in Breast Cancer Patients: Objective Tumour Response and Immunity Indicators

**Figure 4:**
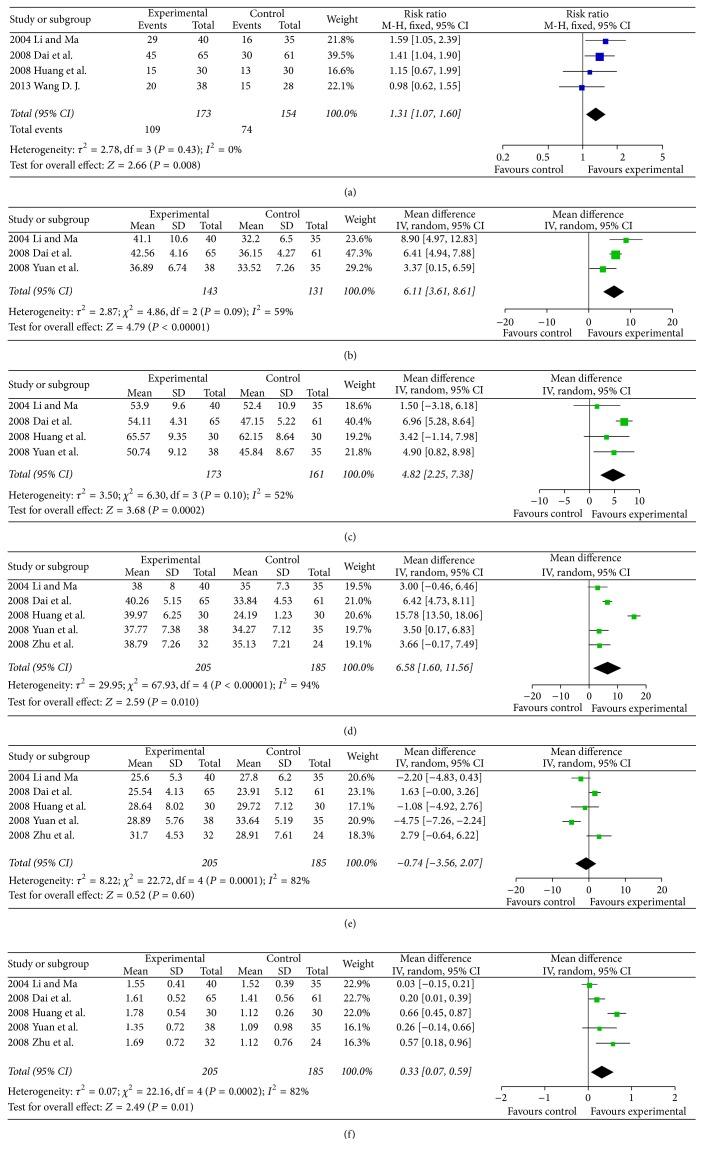
Forest plots of studies comparing Shenqi Fuzheng injection (SQI) invention groups and control groups, measuring the effect of SQI on breast cancer patients including objective tumor response (a) and immunity indicators: NK (b), CD_3_^+^ (c), CD_4_^+^ (d), CD_8_^+^ (e) level, and CD_4_^+^/CD_8_^+^ ratio (f).

Regarding the objective tumour response in breast cancer, there were 4 trials including 173 patients in the SQI intervention group and 154 patients with conventional chemotherapy as control [8, 57, 60, 61]. The objective tumour response in the SQI intervention group was better than control (RR = 1.31, 95% CI 1.07–1.60, *P* = 0.008). The NK level was significantly improved by SQI according to 3 clinical trials [8, 59, 61] which include 143 patients in SQI intervention and 131 patients with conventional chemotherapy (RR = 6.11, 95% CI 3.61–8.61, *P* < 0.00001). Regarding the CD_3_^+^ cell levels, there were 4 clinical trials [8, 59–61] including 173 patients in SQI intervention and 161 patients with conventional chemotherapy The results showed that the SQI intervention was superior to the control in improving the CD_3_^+^ cell levels (RR = 4.82, 95% CI 2.25–7.38, *P* = 0.0002). The CD_4_^+^ cell levels was improved by SQI based on 5 clinical trials [8, 58–61] including 205 patients in the SQI intervention group and 185 patients with conventional chemotherapy (RR = 6.58, 95% CI 1.60–11.56, *P* = 0.010). The CD_4_^+^/CD_8_^+^ ratio was also improved by SQI from the same 5 clinical trials mentioned above [8, 58–61] (RR = 0.33, 95% CI 0.07–0.59, *P* = 0.01). Meanwhile, the CD_8_^+^ cell levels were not significantly decreased by SQI [8, 58–61]. The details were illustrated in Figure 4.

### 3.6. Evaluation of Publication Bias

**Figure 5:**
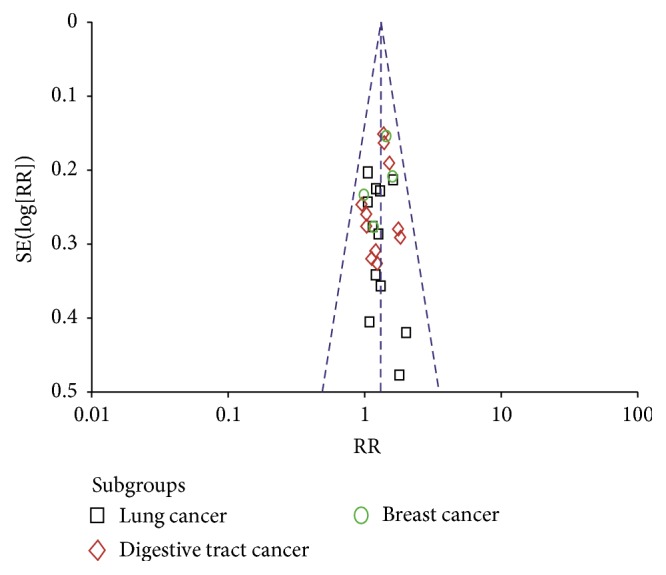
The funnel plot analysis of publication bias on objective tumor response data of lung cancer, digestive tract cancer, and breast cancer patients.

Figure 5 showed the funnel plot based on studies with data on the objective tumour response in lung cancer, digestive tract cancer, and breast cancer patients. The funnel plots indicated asymmetry, which might be due to an insufficient number of trials and significant statistical heterogeneity, suggesting that there might be publication bias.

## 4. Discussion

SQI, a formulation injection made from Chinese medical materials through modern preparation technology, is the representative Chinese medicine formula of nourishing vitality and has been used for adjuvant treatment of lung cancer and gastric cancer since being approved by the SFDA in China in 1999. SQI is given by intravenous drip once per day and initiated three days before chemotherapy. SQI is widely used in clinical practice and had excellent performance from market prospects, achieving sales of 268 million in 2010 and generating approximately 1.3 billion in 2014 [64, 65]. Although its specifications declared that the indications were confined to lung cancer and gastric cancer, other types of cancer patients have been given SQI as a combination drug in the clinic. Its extensive application in the palliative care of cancer was benefited from its definite constitution, stable quality control, and accurate efficacy.

The immune system is the frontline of defense against cancer in human and eliminates cancer cells from normal tissues. Nevertheless, chemotherapy could cause normal function damage by the unselective exhaustion of cancer and normal cells. The activation of immune suppressor mechanisms often appears in cancer patients with chemotherapy [66]. Temporary elimination of IL-10 could overcome the immunosuppressive tumour barrier in mice [67]. The therapeutical potential of the PD-1 and PD-L1 pathway, which is important for T cell regulation in a variety of infectious, autoimmune, and cancer models in mice, was also maximised in recent years. PD-1 knockout mice develop spontaneous autoimmunity [68]. However, the solution for immunosuppression in cancer survivors with chemotherapy remains unsolved but is urgently needed.

The clinical immunoserologic indexes mainly included NK, CD_3_^+^, CD_4_^+^, and CD_8_^+^ levels and CD_4_^+^/CD_8_^+^ ratio. The increases of the NK, CD_3_^+^, CD_4_^+^, and CD_4_^+^/CD_8_^+^ ratio and the decrease of the CD_8_^+^ level showed improvement of immunosuppressive status. It was demonstrated that SQI interventions showed better performance than conventional chemotherapy treatment in terms of improving immunity parameters with enhanced NK, CD_3_^+^, and CD_4_^+^ levels and CD_4_^+^/CD_8_^+^ ratio, suggesting that SQI had a good effect on immune system damage caused by chemotherapy.

Nevertheless, all studies included in the analysis were of low quality according to the Jadad scale. A random allocation was mentioned in all Chinese-language articles; however, the detailed methods of allocation concealment were not described in any articles, which might have led to selection bias and overestimation of the intervention effects. Furthermore, the included trials lacked follow-up outcome indicators to determine the long-term curative effect. The majority of the included trials were classified into three categories: lung cancer, digestive tract cancer, and breast cancer. There were also studies scattered in other cancers such as leukemia and cervical cancer [69–71]. However, those trials were insufficient for conducting a meta-analysis. The meta-analysis of this paper showed comparatively higher heterogeneity for immunity indicators, which might be because the studies included measured different treatment effects under various cancers instead of measuring a single disease effect.

## 5. Conclusions

Although SQI intervention showed immunity enhancement in chemotherapy cancer patients statistically, the meta-analysis results in this paper should be prudently adopted in clinical practice. Although placebo-controlled and double-blinded clinical trials of sizeable samples regarding SQI interventions should be conducted, this meta-analysis still provides useful information for clinical practice.

## Figures and Tables

**Table 1:** Characteristics and quality of patients in included studies.

Study	N (T/C)	Gender (M/F)	Age (years)	Cancer type	Chemotherapy (T&C)	SQI invention (T)	Course	Indicator	Jadad score
Ren 2015 [16]	42/42	T: 24/18;	T: 61.57 ± 5.69;	NSCLC	PP	250 mL, ivgtt, qd, 10d	21d	①②③⑥	2
		C: 25/17	C: 62.53 ± 6.21						
Ren 2014 [20]	65/72	T: 43/22;	T: 66.5 ± 15.3;	NSCLC	TP	50 mL, ivgtt, qd, 24d	21d	②③④⑤	2
		C: 46/26	C: 65.9 ± 14.7						
Wang and Dou 2014 [19]	41/41	T: 31/10;	T: 56.1 ± 4.6;	NSCLC	NP	250 mL, ivgtt, qd, 14d	21d	①③④⑤⑥	2
		C: 29/12	C: 55.7 ± 5.1						
Shan et al. 2014 [21]	40/40	T: 18/22;	T: 58.4 ± 2.1;	NSCLC	DP	250 mL, ivgtt, qd, 14d	21d	①②③④⑤⑥	2
		C: 26/14	C: 58.4 ± 2.1						
Yuan 2014 [18]	35/34	N	N	NSCLC	TP	250 mL, ivgtt, qd, 24d	21d	③④⑤⑥	3
Zhao 2014 [17]	51/51	T: 33/18;	T: 65.08 ± 6.53;	NSCLC	GP	250 mL, ivgtt, qd, 24d	21d	①②	2
		C: 29/22	C: 64.72 ± 6.43						
Wang et al. 2013 [22]	28/28	T: 16/12;	T: 59.14 ± 8.16;	SCLC	DP	250 mL, ivgtt, qd, 14d	21d	①③④⑥	2
		C: 18/10	C: 54.17 ± 9.23						
Li 2012 [24]	25/25	N	55	NSCLC	GP	250 mL, ivgtt, qd, 10d	10d	①	2
Ao et al. 2012 [26]	30/25	N	56	NSCLC	TP	250 mL, ivgtt, qd, 24d	21d	②③④⑤⑥	3
Qiao 2012 [23]	30/30	36/24	61.2	NSCLC	TP	50 mL, ivgtt, qd, 24d	21d	①	2
Ding and Yang 2012 [25]	35/35	T: 20/15;	56.7	NSCLC	TP	250 mL, ivgtt, qd, 21d	21d	①②③④⑤⑥	2
		C: 22/13							
Liu and Ren 2011 [27]	50/50	51/49	57.1	NSCLC	Taxotere & Cisplatin	250 mL, ivgtt, qd, 14d	21d	②③④⑤	2
Liu 2011 [28]	27/27	36/18	62	NSCLC	TP	60 mL, ivgtt, qd, 24d	21d	①	2
Wang 2009 [29]	36/38	T: 23/13;	N	SCLC	DP	250 mL, ivgtt, qd, 28d	21d	①②③⑥	2
		C: 22/16							
Sun et al. 2007 [31]	34/28	T: 21/13;	T: 58;	NSCLC	TP	250 mL, ivgtt, qd, 21d	21d	②③④⑤⑥	2
		C: 20/8	C: 56.5						
Lin and Li 2007 [32]	120/120	N	N	NSCLC	NP/TP	250 mL, ivgtt, qd, 14d	28d	①②⑥	2
Lin 2007 [62]	30/30	T: 18/12;	T: 54.2;	NSCLC	NP	250 mL, ivgtt, qd, 8d	8d	②④⑤⑥	2
		C: 20/10	C: 57.3						
Wang et al. 2007 [30]	28/27	37/12	58.6	NSCLC	NP	250 mL, ivgtt, qd, 21d	21d	①②③④⑤	2
Jiang and Zhuang 2004 [33]	35/32	T: 27/8;	T: 57;	NSCLC	TP	250 mL, ivgtt, qd, 21d	21d	①②③④⑤⑥	2
		C: 26/6	C: 56						
Li 2004 [34]	25/15	T: 15/10;	T: 43;	NSCLC	NP	250 mL, ivgtt, qd, 21d	21d	①②④⑤⑥	2
		C: 10/5	C: 45						
Zhang et al. 2015 [35]	43/43	T: 28/15;	T: 63.5 ± 6.7;	Colon cancer	XELOX	250 mL, ivgtt, qd, 14d	21d	①②④⑤⑥	2
		C: 29/14	C: 64.3 ± 7.2						
Wen et al. 2014 [63]	15/15	T: 12/3;	T: 59.9 ± 7.7;	Gastric cancer	FOLFOX4	250 mL, ivgtt, qd, 10d	14d	①	2
		C: 11/4	C: 59.6 ± 5.6						
Yan et al. 2014 [36]	56/56	T: 33/23;	T: 56.2 ± 11.3;	Colon cancer	FOLFOX4	250 mL, ivgtt, qd, 5d	14d	③④⑤⑥	2
		C: 35/21	C: 56.9 ± 10.8						
Wen 2014 [37]	23/23	T: 18/5;	66	Gastric cancer	XELOX	250 mL, ivgtt, qd, 10d	21d	①	2
		C: 16/7							
Wang 2014 [38]	42/42	T: 23/19;	T: 64.2 ± 11.3;	Gastric cancer	FOLFOX4	250 mL, ivgtt, qd, 14d	28d	①	2
		C: 22/20	C: 65.9 ± 3.4						
Han et al. 2014 [39]	34/34	38/30	52.6 ± 4.12	Gastric cancer	FOLFOX6	250 mL, ivgtt, qd, 21d	21d	①③④⑤⑥	2
Wang 2013 [41]	38/38	T: 25/13;	T: 53.6 ± 15.8;	Gastrointestinal cancer	DF	250 mL, ivgtt, qd, 21d	21d	①	2
		C: 24/14	C: 55.3 ± 16.2						
Tan et al. 2013 [42]	20/20	28/12	64	Colon cancer	XELOX	250 mL, ivgtt, qd, 14d	21d	①②④⑤⑥	2
Jin 2013 [43]	40/40	T: 24/16;	T: 45.0 ± 12.5;	Gastric cancer	Oxaliplatin & 5-Fu	250 mL, ivgtt, qd, 5d	5d	①②③④⑤⑥	2
		C: 23/17	C: 44.8 ± 12.5						
Yin and Jiang 2013 [40]	26/27	T: 14/12;	59	Gastric cancer	SP	250 mL, ivgtt, qd, 24d	21d	①②③④⑤⑥	2
		C: 13/14							
Huajun and Xinmei 2012 [45]	28/28	33/23	47.5 ± 3.2	Gastrointestinal cancer	FOLFOX	250 mL, ivgtt, qd, 21d	21d	③④⑤⑥	2
Ren and Wang 2012 [44]	33/32	30/35	62	Gastric cancer	FOLFOX4	250 mL, ivgtt, qd, 14d	14d	①	3
Liu and Han 2011 [46]	45/40	T: 25/20;	T: 64.8 ± 7.0;	Gastric cancer	FOLFOX4	250 mL, ivgtt, qd, 28d	28d	②③④⑤⑥	2
		C: 21/19	C: 65.1 ± 6.9						
Guo et al. 2011 [47]	30/24	N	65.4	Colorectal cancer	Oxaliplatin & 5-Fu	250 mL, ivgtt, qd, 7d	14d	①②③④⑥	2
Zhang et al. 2010 [48]	20/20	T: 12/8;	T: 48.5 ± 12.8;	Colorectal cancer	FOLFOX	250 mL, ivgtt, qd, 5d	5d	④	2
		C: 11/9	C: 47.6 ± 11.9						
Wang 2010 [50]	30/30	T: 25/5;	T: 58.0 ± 2.9;	Gastrointestinal cancer	Oxaliplatin & 5-Fu	250 mL, ivgtt, qd, 14d	14d	③④⑤⑥	2
		C: 24/6	C: 58.7 ± 2.6						
Xu 2010 [49]	30/30	T: 24/6;	57	esophageal cancer	PF	250 mL, ivgtt, qd, 29d	28d	④⑤⑥	2
		C: 26/4							
Liang et al. 2009 [54]	76/76	T: 50/26;	53	Colorectal cancer	FOLFOX	250 mL, ivgtt, qd, 10d	21d	①⑥	3
		C: 51/25							
Ni et al. 2009 [52]	70/65	T: 44/26;	59	Colorectal cancer	FOLFOX	250 mL, ivgtt, qd, 17d	14d	③④⑤⑥	2
		C: 42/23							
Zhang et al. 2009 [51]	40/36	N	56.3	Colon cancer	FOLFOX4	250 mL, ivgtt, qd, 7d	14d	④⑤⑥	2
Liu and Gong 2009 [53]	30/30	38/22	62.5	Gastric cancer	Oxaliplatin & 5-Fu	250 mL, ivgtt, qd, 14d	14d	④⑤⑥	2
Wang et al. 2008 [55]	40/40	T: 22/18;	T: 57.34 ± 16;	Gastrointestinal cancer	FOLFOX6	250 mL, ivgtt, qd, 7d	14d	④⑤⑥	2
		C: 22/18	C: 57.44 ± 16						
Sun et al. 2002 [56]	46/32	45/32	49.6	Gastrointestinal cancer	Oxaliplatin & 5-Fu	250 mL, ivgtt, qd, 21d	21d	①③④⑥	2
Wang 2013 [57]	38/38	0/76	T: 45.5 ± 9.8;	Breast cacer	CAF	250 mL, ivgtt, qd, 14d	21d	①	2
			C: 45.2 ± 9.8						
Yuan et al. 2008 [59]	38/35	0/73	N	Breast cacer	CAF	250 mL, ivgtt, qd, 20d	20d	②③④⑤⑥	2
Zhu et al. 2008 [58]	32/24	0/56	52.5	Breast cacer	CEF	250 mL, ivgtt, qd, 10d	21d	④⑤⑥	2
Huang et al. 2008 [60]	30/30	0/60	47	Breast cacer	CTF	250 mL, ivgtt, qd, 21d	21d	①③④⑤⑥	3
Dai et al. 2008 [8]	65/65	0/130	T: 45.5 ± 26.8;	Breast cacer	CEF	250 mL, ivgtt, qd, 21d	21d	①②③④⑤⑥	2
			C: 46.1 ± 27.5						
Li and Ma 2004 [61]	40/35	0/75	5.46	Breast cacer	NE	250 mL, ivgtt, qd, 10d	28d	①②③④⑤⑥	2

T: the trials where a SQI intervention was conducted; C: the control groups of patients with regular chemotherapy. NSCLC: non-smalll cell lung cancer; SCLC: smalll cell lung cancer; PP: pemetrexed disodium & cisplatine; TP: taxol & cisplatin; NP: navelbine & cisplatin; DP: docetaxel & cisplatin; GP: gemcitabine & cisplatin; XELOX: oxaliplatin and capecitabine; FOLFOX: oxaliplatin, leucovorin calcium and fluorouracil; DF: cisplatin, leucovorin calcium and 5-Fu; SP: cisplatin and fluorouracil derivant; PF: cisplatin and 5-Fu; CAF: cyclophosphamide, adriamycin and fluorouracil; CEF: cyclophosphamide, epirubicin and fluorouracil; CTF: cyclophosphamide, pirarubicin and 5-Fu; NE: navelbine and epirubicin. ^①^objective tumor response; ^②^natural killer cell (NK)level; ^③^matured T lymphocytes (CD_3_^+^) cell level; ^④^inducer lymphocyte/helper T lymphocyte (CD_4_^+^) level; ^⑤^suppressor T cell/cytotoxic T cell (CD_8_^+^) cell level; ^⑥^CD_4_^+^/CD_8_^+^ ratio.

**Table 2:** The detailed safety evaluation outcome in combination medication of SQI and chemotherapy agents.

Cancer	Gastrointestinal reaction			Routine blood indexes					LI	RI	KPS	Peripheral nerve toxicity	Oral ulcer	Hair loss	Fever	Phlebitis	HFS
	NV	Anorexia	Diarrhea	WBC**↓**	RBC**↓**	HGB**↓**	PLT**↓**	NEU**↓**									
Lung cancer	8 [16, 19, 20, 23, 24, 28–30]	5 [13, 14, 30, 33, 34]	6 [16, 19, 20, 23, 29, 30]	13 [16, 18–20, 22, 23, 26, 28–31, 33, 34]	N	11 [19, 20, 22–24, 26, 28–31, 33]	11 [19, 20, 22–24, 26, 28–31, 33]	N	1 [22]	2 [16, 22]	10 [17–19, 21, 22, 24, 29, 30, 32, 33]	1 [20]	N	N	2 [16, 28]	N	N
Digestive tract cancer	14 [37–39, 41, 43, 44, 46–50, 52–54]	5 [38, 41, 43, 44, 48]	10 [38, 41, 43, 44, 46–49, 53, 54]	12 [36, 37, 40, 46, 47, 49–54, 56]	1 [36]	6 [36, 37, 40, 49, 53, 56]	12 [36, 37, 39, 40, 46, 47, 49, 51–54, 56]	N	11 [37, 43, 44, 46–52, 54]	8 [37, 43, 44, 46, 48, 49, 52, 54]	10 [35–37, 42, 46, 47, 49, 50, 52, 54]	4 [38, 39, 47, 54]	2 [46, 54]	3 [43, 46, 54]	2 [46, 52]	N	2 [43, 46]
Breast cancer	3 [8, 57, 61]	1 [8]	2 [8, 61]	5 [8, 57, 58, 60, 61]	N	3 [8, 57, 58]	3 [8, 58, 61]	N	2 [57, 61]	2 [57, 61]	3 [57, 60, 61]	N	N	2 [57, 61]	N	1 [61]	N

NV: nausea and vomiting; LI: liver injury; RI: renal injury; KPS: Karnofsky performance score; WBC: white blood cell; RBC: red blood cell; HGB: hemoglobin; PLT: platelet; NEU: neutrophil; HFS: hand-foot syndrome; N: not mentioned.
